# Case Report: Synovial chondromatosis in sport climbers fingers

**DOI:** 10.3389/fspor.2025.1513112

**Published:** 2025-02-18

**Authors:** Helmut Becker, Xeber Iruretagoiena-Urbieta, Volker Schöffl

**Affiliations:** ^1^ Redpoint Physiotherapy, Calgary, AB, Canada; ^2^ Deusto Physical TherapIker, Physical Therapy Department, Faculty of Health Sciences, University of Deusto, San Sebastian, Spain; ^3^ Physiotherapy, Upper Limb Unit, Eskura Osasun Zentroa, Beasain, Spain; ^4^ Sputnik Investigación, Madrid, Spain; ^5^ Department of Orthopedic and Trauma Surgery, Klinikum Bamberg, Bamberg, Germany; ^6^ Department of Orthopedic and Trauma Surgery, Friedrich Alexander University Erlangen-Nuremberg, Erlangen, Germany; ^7^ School of Health, Leeds Becket University, Leeds, United Kingdom; ^8^ Section of Wilderness Medicine, Department of Emergency Medicine, School of Medicine, University of Colorado, Denver, CO, United States, United States; ^9^ Division of Exercise Physiology and Metabolism, Department of Sport Science, University of Bayreuth, Bayreuth, Germany

**Keywords:** chondromatosis, climbing, finger, rock climbing, finger injury

## Abstract

**Introduction:**

Sport Climbing is a rapidly developing sport with an increasing variety and number of injuries, especially now that it is an Olympic event. The objective of this study is to firstly report the presence of Finger Synovial Chondromatosis (FSC) found in the fingers of climbers. Synovial chondromatosis is a rare condition and particularly rare in the hands and fingers.

**Methods:**

We prospectively evaluated all climbers with finger injuries presenting in 2022 for the presence of FSC in the finger joints. 13 patients in this case series were included with complaints of finger pain for more than 6 weeks. The study was performed in our Sports Medical Centre in Bamberg, Germany. The diagnosis was made based on in person clinical assessment, ultrasound examination and additional radiological findings (e.g., x-ray, CT, MRI) as applicable.

**Results:**

We detected 13 male climbers with FSC with experience ranging from intermediate to elite level. 77% (10/13) of the patients did not complain of any symptoms of the FSC and presented with other diagnoses (e.g.pulley rupture, tenosynovitis) and the finding of FSC was just an additional finding. In 12 subjects FSC was found in the proximal interphalangeal (PIP) joint and one in the metacarpo-pahalangeal (MCP) joint and distributed as follows: right (R) side 69%, left (L) side 38%, considering 15% in both hands. The prevalence corresponded to the digits are as follows: D2 15%, D3 77%, and D4 15%.

**Discussion:**

With the rapid development in sport climbing there has been a rise in the intensity, volume and variety of training, which may be the key factors contributing to the spectrum of injuries associated with climbing. Hand and finger climbing related injuries are well documented, however the incidental discovery of this rare condition warrants its inclusion as another differential diagnosis in the spectrum of climbing related pathologies. At the moment, not enough is understood about FSC and its long-term consequences therefore further analysis is warranted for future studies.

## Introduction

Sport Climbing is a rapidly developing sport, particularly in recent years after becoming an Olympic event (1, 2). Accordingly, the variety of injuries is also increasing (1, 2). Overall, injuries in climbing generally affect the upper extremities, with the leading diagnosis of tenosynovitis of the fingers, finger pulley injuries, capsulitis of the finger joints and growth plate injuries of the fingers (1–3). While a lot of research has already been conducted on these injuries, we present a new pathology that has not been previously described for the climbing community, in which we call Finger Synovial Chondromatosis (FSC).

Synovial chondromatosis (SC) is generally considered to be a rare process that can affect both men and women, and has been described in the literature by various authors through case reports only (4–15). It presents as a primary or secondary condition as a result of an articular abnormality and is reported to typically affect large joints such as the knees, hips, elbows and shoulders (4–15). It is most common between the third and fifth decades of life (4, 5, 15–18). Primary SC is characterized by cartilage metaplasia, typically of unknown etiology, resulting in multiple intra-articular and periarticular loose osteocartilaginous bodies. It is a rare benign proliferative disease of the joint synovium, tenosynovium, or bursal lining (4–7, 9). Secondary SC is associated with mechanical joint abnormalities, injuries, or arthritis that cause intra articular chondral bodies (8). SC of the joints in the hands, accounting for about 2% of all cases (6, 9), is considered extremely rare (4, 6, 7, 9–12). Although rare, another form of chondromatosis, an extra-articular tenosynovial chondromatosis is common in the hands and feet (5, 7, 8, 10–12). Additionally, in the hands, it presents typically in the tendon sheath of the digits, with the flexor tendons slightly more commonly involved than the extensor tendons (5, 7–9, 11, 12). SC may be presented in the articular cartilage or synovium, which is differentiated from tenosynovium (4–12). Although they resemble in their histological presentation and morphology, they differ regarding their pathophysiology as FSC originates from the joint (7–10). The most common symptoms of articular SC reported in the literature are persistent pain, cases of local tenderness, swelling and limited range of motion (4–12). This process may present mildly symptomatic or asymptomatic, and in many cases without severely affecting activities of daily living (4–15).

The aim of this work is, firstly, to present the pathology of FSC in sport climbers and to explore whether these findings are random or indicate a clinical pathology.

## Methods

We prospectively evaluated sport climbers with finger injuries presenting to our Sports Medical Clinic in Bamberg, Germany, in 2022 for the presence of FSC in the finger joints. Within that period we saw 13 cases of FSC on the fingers included in this case series collected from a population within the Bavarian region in Germany. This sample size represents less than 3% of the overall patient population with climbing related injuries that are treated at this specific Sports Medical Clinic each year.

All climbing patients included in the study who complained of finger pain for more than six weeks came for assessment and treatment at our Sports Medical Centre in Bamberg, Germany, in 2022. The study was approved by the institutional ethical board and all patients provided informed consent.

The diagnosis was reached based on in person clinical assessment and further investigation including, ultrasound examination (US) and other radiological findings, such as plain Radiographs (x-ray), Computer Tomography (CT), and Magnetic Resonance Imaging (MRI) as applicable. The US examination was used in every case in this study, in which we used a 18 Mhz hockey stick linear transducer with a LOGIQ™ ultrasound unit (GE HealthCare, Chicago, Illinois, USA). Abundant US gel was used to avoid compression of the finger by the transducer. The finger examination position was 0° or neutral position of the metacarpophalangeal (MCP), proximal interphalangeal (PIP), and distal interphalangeal (DIP) joints with the hand in supine position.

The differential diagnosis for other possible conditions were considered to be ruled out for adequate discernment. Final diagnosis was reviewed and confirmed by the main author, who is a board-certified orthopaedic surgeon with more than 25 years of experience with climbing injuries and their ultrasound evaluation based on the differential diagnosis criteria of climbing injuries as stated in the literature (16–18). A standard questionnaire and examination protocol was conducted in person and during in clinic consultation. Only patients suffering from pain during or after sport climbing or bouldering were included in the study. Prior injury or surgery at the respective finger was an exclusion criteria as well as any systemic disease or permanent medication. We identified 13 cases within our sport climbing patients to our climbing specific clinic. All patients who were diagnosed with FSC were included. Statistical analysis was performed using SPSS 22.0 software (SPSS Science, Chicago, IL, USA) for data collection. The statistical analysis was performed by the authors. All measured values are reported as means and standard deviations.

## Results

All patients identified with FSC were male and the general descriptions of the biometric data and climbing experience are listed on Table 1. None of them reported the use of any medication, and 23% (3/13) reported using additional supplements, mostly Glucosamine and Chondroitin, Vitamin complex, and Creatine. No other relevant medical condition was reported.

**Table 1:** Demographics of climbers with FSC.

Classifications	All subjects mean ± SD
Number of subjects	13
Age	37 ± 10
Range	26–57
Height (cm)	177.6 ± 5.5
Weight (Kg)	70.5 ± 8.6
Climbing experience	
Climbing years	19.1 ± 10.6
Range	0.5–40
Climbing Level UIAA metric	9.33 ± 1.09
Range	7.33–10.66
Bouldering years	18.1 ± 10.5
Range	3–36
Bouldering level (V scale)	9 ± 3.2
Range	5–14
Climbing/bouldering hours/week	11.5 ± 8.7
Range	4–17
Dedicated climbing modality	
Sport climbing	47.9 ± 34.6%
Bouldering	48.9 ± 33.5%
Alpine/traditional	1.9 ± 5.6%
Competition	
Yes	31% (4/13)
No	69% (9/13)
Warm Up routine	77% (10/13)
Additional strength training	54% (7/13)
Hours/week strength training	2.1 ± 2.7
Range	0–6
Campus board (CB) training	46% (6/13)
Hours/week	0.6 ± 1
Range	0.5–2
Additional weight training	15% (2/13)
Hrs/week	0.2 ± 0.6
Range	0.5–2
Preferred grip technique	
Close crimp	38% (5/13)
Half crimp	15% (2/13)
Open grip	31% (4/13)
No answer	15% (2/13)
Other sports practice	77% (10/13)
Hours/week	2.4 ± 2.3
Range	1 -10
Compensatory training (%)	62% (8/13)
Hrs/week	0.9 ± 1.4
Range	0.5–4

The diagnostic findings and final diagnosis are depicted in Table 2. The location of the lesion is defined by the side as right or left (R or L), the particular digit (D1-5) and which region of the phalanx (phlx) including the associated joint: proximal interphalangeal, distal interphalangeal, and metacarpophalangeal (PIP, DIP, MCP) respectively. The presence of FSC in the fingers in 12 subjects was in the PIP joint and one in the MCP joint. The site of FSC presence in each of the 13 participants was also the site of the chief complaint that had brought each participant to seek a clinical consultation. The prevalence of which side was affected are as follows: R side 69% (9/13), L side 38% (5/13), considering 15% (2/13) in both hands. The prevalence corresponded to the digits are as follows: D2 15% (2/13), D3 77% (10/13), and D4 15% (2/13). 23% of the participants (3/13) reported previous injuries onto the finger. 77% (10/13) of the patients did not complain of any symptoms of the FSC and presented with other diagnoses (e.g.,pulley rupture, tenosynovitis) and the finding of FSC was just an additional random finding. In 2 cases FSC was found bilaterally. Other diagnoses with additionally random finding of FSC were joint capsulitis 38% (5/13), pulley ruptures of A2 8% (1/13), and A4 8% (1/13), and a case of bone spurs 8% (1/13). The detailed information is given in Table 2.

**Table 2:** Diagnostic findings in all subjects.

N	Location: side/finger/joint/phalanx	Symptom: yes or no	Diagnostic imaging	Final diagnosis	Treatment
1	R D2 PIP head of proximal phlx	Y	x-Ray, US, MRI, CT	Synovial Chondromatosis	Conservative
2	Bilat D3 PIP head of proximal phlx	N	US, MRI	Synovial Chondromatosis and PIP Capsulitis	Conservative
3	R D3 MCP head of proximal phlx	N	US, CT	Synovial Chondromatosis and MCP Synovitis and Capsulitis	Conservative
4	R D3 PIP head of proximal phlx	N	US	Synovial Chondromatosis and PIP Capsulitis	Conservative
5	R D4 PIP head of proximal phlx	N	US	Synovial Chondromatosis and A4 Pulley Rupture	Conservative
6	R D3 PIP head of proximal phlx	N	US	Synovial Chondromatosis and Synovitis	Conservative
7	L D2 PIP head of proximal phlx	Y	US	Synovial Chondromatosis and PIP Capsulitis	PRP
8	R D3 PIP head of proximal phlx	N	US, MRI	Synovial Chondromatosis and A2 Pulley Rupture	Conservative
9	R D3 PIP base of 2nd and head of proximal phlx	N	US	Synovial Chondromatosis and Tenosynovitis	Conservative
10	R D3 PIP head of proximal phlx	Y	US	Synovial Chondromatosis and PIP Capsulitis	Conservative
11	L D3 PIP head of proximal phlx	N	US	Synovial Chondromatosis	Conservative
12	L D3 PIP head of proximal phlx and R D4 PIP head of proximal phlx	N	US	Bilateral Asymmetrical Synovial Chondromatosis	Conservative
13	L D3 PIP base of middle phlx	N	US	Synovial Chondromatosis and Bone Spur and Pulley Rupture	Conservative

The diagnostic imaging methods used to detect the presence of FSC in this study was as follows: Ultrasound (US) 100% (13/13), Magnetic Resonance Imaging (MRI) 23% (3/13), Computed Tomography (CT) in 2 cases, and x-Rays in one case.

None of the patients required any specific mode of therapy for FSC afterwards considering the clinical findings and functional status. No surgical procedures were performed in this case series as per surgeon's discretion based on the functional and clinical presentation of the subjects.

## Discussion

The primary objective of this study is to report the presence of FSC found in climbers. Overall SC is a rare manifestation, particularly in the hands, and is typically not associated with a history of trauma (4–12). Hand and finger injuries are well documented to be the most common amongst climbers (1–3, 16–29), with the middle and ring fingers most frequently affected (1–3, 19, 20, 22, 25). As previously mentioned, the most common injuries within the fingers are tenosynovitis of the flexor tendons, pulley injuries, capsulitis and also growth plate injuries of the fingers (primarily in adolescents), which are believed to be caused by repetitive stress and microtrauma rather than from a single acute event (3, 19, 20, 25). The rapid development in sport climbing in general has been attributed to the intensity, volume and variety of training, which may be the key factors contributing to the spectrum of injuries associated with climbing (19). Therefore, it is reasonable to assume that other new structurally related lesions of the hands and fingers may also become more prevalent over time as climbing activity expands worldwide (1).

The ability to grip on holds is paramount in order to perform rock climbing. Technically, most gripping positions pose more stress upon the flexor mechanism of the hands and fingers. Since this case series study relates specifically to finger injuries in climbing, we also asked the participants to report their preferred gripping position as described and differentiated in Lutter at al (1). In our study group, 11/13 (85%) of the participants were able to specify their preferred gripping position, and it was ranked from most to least preferred as follows: closed crimp 38%(*n* = 5), open grip 31%(*n* = 4), and half crimp 15% (*n* = 2). A correlation to certain repetitive gripping positions and increased loads to particularly common finger injuries have been established in previous studies (1, 3, 19–21). Based on the few cases of FSC it is not possible yet to correlate the injury to any specific preferred hand position. Considering what it is broadly understood in other studies regarding hand and finger injuries in climbers, it may be reasonable to consider FSC to be a manifestation of secondary SC due to onset of osteoarthritis, as we know that osteoarthritis is one consequence of the closed crimp grip position for example.

Looking at our patient group, this sample it is vastly focused in the sport climbing, including bouldering with 77% (N 10/13) of the participants climbing for 10 years or more and at least at a UIAA metric level of 9 (UIAA 9, French 7c/7c+, YDS 5.12d) in sport climbing but with also a wide range in the bouldering V scale (9 ± 3.2) (24). This spectrum of experience represents what can be considered of a relatively experienced climber to a world elite level climber. It is interesting that all climbers in this sample with FSC have a long history of performing the sport, hence the condition may be seen as a long-term adjustment to the mechanical load rather than as an acute consequence.

Surprisingly all participants in this study are male considering the fact that SC overall is also present in females as well (4–12). One explanation may be that we see a higher portion of male climbers than females and they tend to exert harder (30), thus placing more stress onto the fingers. Also, males tend to be heavier in mass, consequently posing heavier loads over the fingers (30). Additionally, the mean age of our participants is 37, which correlates to the documented period in the literature about SC being most common between the third and fifth decades of life (4, 5, 12–15). However, considering that such finding occurred even in a 26 yr old subject, a rather young age for its typical presentation, it highlights the attention towards its inclusion as a differential diagnosis when clinicians evaluate hand and finger injuries in adult climbers of all ages. Understandably, our sample size is small and limited to males and from a particular region within Europe, therefore learning more about FSC presence in climbers, a growing population, may provide physicians a differential diagnosis discerning from pulley injuries, capsular or ligamentous lesions and its possible associated cysts or scar formations within the fingers (22).

Evidence of SC in athletes in the literature is also limited and typically reported presenting in large joints (13–15). SC was only once reported about in rock climbers (25), although not thoroughly investigated yet. All participants in our study group were diagnosed with a condition in the same site in which FSC was found. In this study particularly, the site of FSC was found in the volar aspect of the head of the proximal phalanx in the PIP joint in all cases, and only 2 (15.4%) presented at the base of the middle phalanx. While the literature describes the prevalence of SC typically present within the digits in the PIP, MCP and also DIP (4–12). Again, it is probably too early to speculate with certainty whether or not there is a direct correlation between these findings of FSC and the typical onset of injury in climbers, based on the overall anatomical proximity of the several structures within the digits. The most common symptoms of articular SC reported in the literature are persistent pain, cases of local tenderness, swelling and limited range of motion (4–12). However, 11 of the 13 subjects in our study did not report any symptoms in this regard and were referred to the sports medicine center for other hand and finger-related symptoms associated with the practice of climbing. Therefore, the discovery of FSC in our participants is incidental in nature, perhaps due to the lack of a specific illness script. As a reasonable consideration, the symptom presentation of several conditions within the hand and fingers, including the ones found in our subjects, overlap in some aspects with the symptoms that FSC may present by itself, which warrants attention within the spectrum of a differential diagnosis amongst the climbing community.

As for management, the preferred method of treatment found in the literature of SC is surgical resection (4–15), because the masses found were rather typically large. On the contrary this approach was not warranted for our 13 subjects, as these patients either presented no symptoms specifically associated with FSC at all or responded well to a conservative therapy—which included local steroid injection, clinical advice about rest, icing, physiotherapy attendance as felt needed, mobility exercises, management of climbing style and stress reduction, terrain and frequency. No specific rehabilitation protocol was administered specifically for this participant sample considering that clinical care in fact was focused on the primary diagnoses for each case and not FSC. This study did not intend to explore specifically treatment protocols for the found FSC, because the condition is not fully understood yet, which may constitute a limitation here. As more studies are developed overtime with broader explanations for how FSC may develop and how it interacts with the presence of other already understood conditions, we suggest that it will be possible to develop more targeted treatment management protocols.

Considering that 38% (*n* = 5) of our subjects presented with the adjunct diagnosis of joint capsulitis in the finger, and that its presence in the PIP is the third most frequent injury in climbers, accounting for 6%–10% of all climbing injuries (19), we believe that it may deserve further analysis. This injury pattern can be caused by high load during closed crimp position with DIP joint into hyperextension generating high peak of pressure within the PIP capsule (21). It can also occur as secondary to acute injuries, such as collateral ligament sprains or chronic repetitive microtrauma, thus hypothetically developing FSC. Therefore, even if FSC may present as asymptomatic, further research may determine its relation with joint capsulitis.

**Figure 1:**
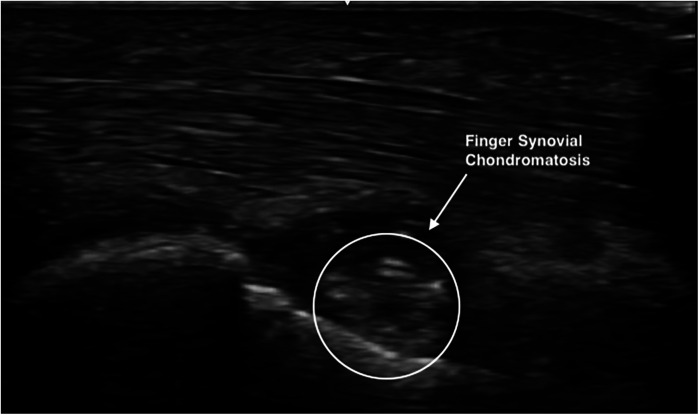
US of the R D3 MCP head of 1st phalanx.

**Figure 2:**
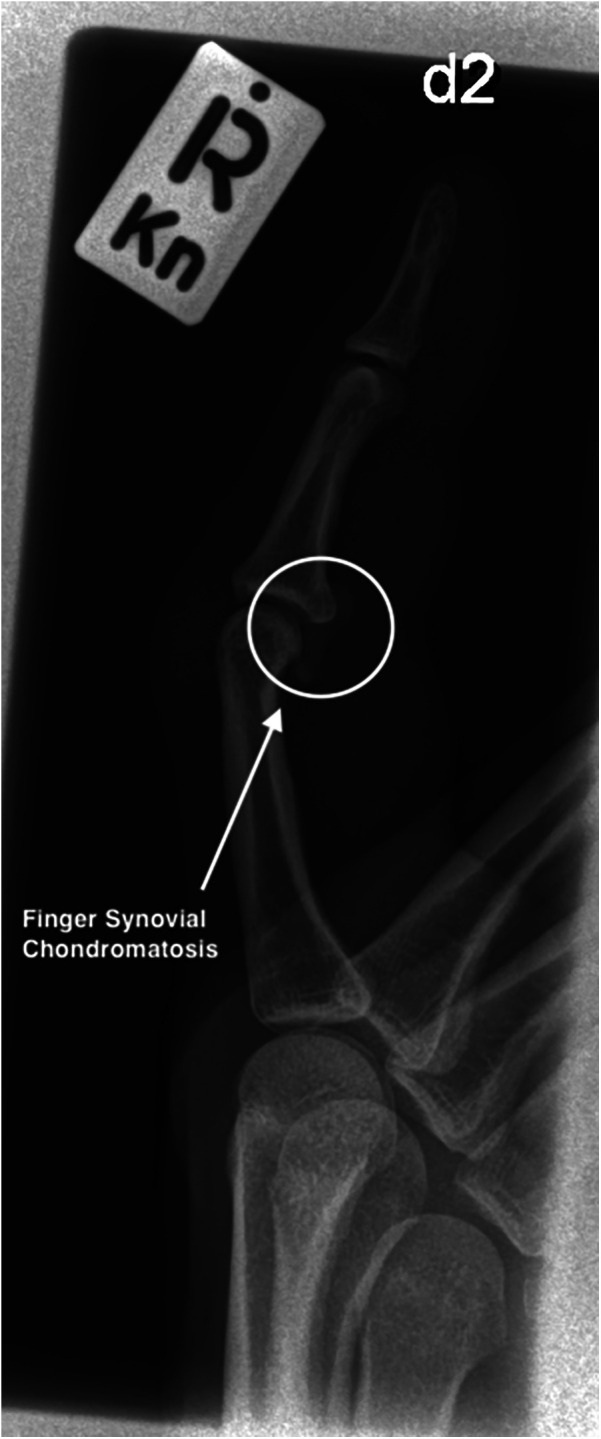
X-ray of the R D2 PIP head of 1st phalanx.

Regarding the diagnostic ability to detect the presence of FSC through imaging examination, the US exam demonstrated reasonable accuracy as a viable and inexpensive method (Figure 1). In a systematic review by Iruretagoiena-Urbieta et al., a conclusion was reached regarding the US examination of finger related climbing injuries, is that today it is considered the gold standard for a number of reasons, which includes cost effectiveness, accessibility and non-invasive method. Comparative US and MRI study detected 98% sensitivity and 100% specificity for US regarding pulley injuries in climbers (26). Additionally, it has recently been observed that the US is a valid and reliable tool for identifying anatomical landmarks for finger injuries in climbers (27). As FSC becomes better understood, specific US protocols can be developed in order to model more adequately such differential diagnosis. Not all clinical settings are able to readily offer the use of MRI in the pursuit of such a rare condition. Also, in our 2 cases who had an MRI exam, FSC was only depicted in one subject, while in the other it was only depicted in the US exam. The use of x-Rays has also been widely utilized in other studies involving SC in the fingers identifying the calcified masses (4–12). Therefore, it may also be possible to standardize the use of x-rays in future studies, as this is a tool easily accessible (Figure 2).

The main study limitations included the fact that no surgical excision was performed, and consequently absence of histological studies compared to other case studies in SC. In spite of the fact that these were not performed because the cases didn't require them, it would have been beneficial and complementary in order to confirm our hypothesis of FSC diagnosis within our subjects. Although US exam findings are technically operator dependent, which can constitute a limitation in this study, it is still a convenient and highly accurate method to identify several hand and finger related conditions that concern the vast majority of injured climbers (28), such as pulley tears (16–19, 29), and yet a cost effective method to identify SC within the hand or fingers compared to MRI or CT (7). While this study is limited to a sample collected in the Bavarian region of Germany, it is important to consider that sport climbing represents a strong practice there, which includes the world renowned Frankenjura. That is also represented by the significant amount of consultations regarding climbing specific injuries yearly by the Sports Medical Clinic in Bamberg for over two decades.

There is much to be learned about SC in general, how it is manifested, how it interacts with other structures and its long-term effects, but also how to adequately manage its treatment. FSC as we described in this study, is a newly identified process in the climber's pathological spectrum of presentations of hand and finger related injuries. Considering the rapid expansion of sport climbing and its variety of hand and finger related injuries, adequate exploration of the possible differential diagnosis is important for the longevity of the climber's hands health. Learning about the pathology of such a rare condition as FSC is important so no misdiagnose takes place in light of proper treatment. Even though our study group did not require further surgical interventions, and were managed with non-invasive conservative therapy, we don't know yet the long-term implications of this clinical presentation. Climbing can cause extreme exertions of the hand and finger structures possibly in similar fashion to various other activities. For example, other hand and gripping dominant athletes or heavy-duty workers which also suffer from hand injuries and could potentially be developing FSC unidentified, and not documented in other studies yet. At the moment, not enough is understood about FSC and its long-term consequences therefore further analysis is warranted for future studies.

## Data Availability

The datasets presented in this article are not readily available because Patient Confidentiality. Requests to access the datasets should be directed to helmut@redpointphysio.com.
